# Prevalence estimates of the insulin resistance and associated prevalence of heart failure among United Status adults

**DOI:** 10.1186/s12872-023-03294-9

**Published:** 2023-06-10

**Authors:** Xiaozhong Li, Jihong Wang, Liyan Niu, Ziqi Tan, Jianyong Ma, Ling He, Peng Yu, Xiao Liu, Juxiang Li

**Affiliations:** 1 Department of Cardiovascular Medicine, the Second Affiliated Hospital of Nanchang University, Nanchang, 330006 China; 2 Department of Pharmacology and Systems Physiology, University of Cincinnati College of Medicine, Cincinnati, OH 45267 USA; 3 Department of Geriatrics Medicine, the Second Affiliated Hospital of Nanchang University, Nanchang, 330006 China; 4 Department of Endocrinology Medicine, the Second Affiliated Hospital of Nanchang University, Nanchang, 330006 China; 5 Department of Cardiology, Sun Yat-Sen Memorial Hospital of Sun Yat-Sen University, Guangzhou, 510080 Guangdong China; 6 Guangzhou Key Laboratory of Molecular Mechanism and Translation in Major Cardiovascular Disease, Guangzhou, 510120 Guangdong China

**Keywords:** Triglyceride glucose index, Homeostatic model assessment of insulin resistance, Insulin resistance, Heart failure, Nation health and nutrition examination survey

## Abstract

**Background:**

The triglyceride glucose (TyG) index, a metric for estimating insulin resistance (IR), is linked with cardiovascular disease (CVD) morbidity and mortality among the population regardless of diabetic status. However, IR prevalence and the association between the TyG index and heart failure (HF) in Americans is unclear.

**Methods:**

The Nation Health and Nutrition Examination Survey (NHANES) (2009–2018) dataset was used. IR was defined by homeostatic model assessment of insulin resistance (HOMA-IR) > 2.0 and 1.5. The TyG index was calculated as Ln [fasting triglycerides (mg/dL) × fasting glucose (mg/dL)/2]. A weighted logistic regression was applied to evaluate the association between the TyG index and the prevalence of HF.

**Results:**

This study comprised 12,388 people, including 322 (2.6%) individuals with HF. The average prevalence of IR was found to be 13.9% and 22.7% for cutoff values greater than 2.0 and 1.5, respectively. HOMA-IR and the TyG index showed a moderate correlation (*r* = 0.30). There is a significant positive association between the TyG index and HF prevalence (per 1-unit increment; adjusted OR [aOR]: 1.34; 95% confidence interval [CI]: 1.02–1.76). Patients with higher TyG values were associated with a prevalence of HF (OR:1.41; 95% CI: 1.01,1.95) (quartiles 4 vs 1–3). The TyG index is associated with a higher prevalence of dyslipidemia, coronary heart disease, and hypertension but not a stroke (cerebrovascular disease).

**Conclusions:**

Our results show that IR does not considerably increase from 2008 to 2018 in American adults. A moderate correlation is noted between HOMA-IR and the TyG index. TyG index is associated with the prevalence of HF, as were other cardiovascular diseases.

**Supplementary Information:**

The online version contains supplementary material available at 10.1186/s12872-023-03294-9.

## Introduction

The homeostatic model assessment of insulin resistance (HOMA-IR) and the triglyceride glucose index (TyG index) are regarded as markers of insulin resistance (IR). As a circumstantial marker of IR, HOMA-IR is a relatively widely used approach in practice [1]; however, it is costly and unavailable in most laboratories in developing countries. The TyG index, which is considered a replacement marker of IR by emerging studies [2, 3], was calculated applying the formula Ln [fasting triglycerides (mg/dL) × fasting glucose (mg/dL)/2]. Thus, the TyG index is less expensive and easily available in clinical practice. Current studies have discovered an established association between the TyG index and several diseases, including hypertension [4], carotid atherosclerosis [5], stroke [6], CVD [7], and coronary artery stenosis [8]. However, in Americans, studies on the prevalence and associated cardiovascular risk of the TyG index are limited [9, 10].

The end stage of the majority of cardiovascular diseases is heart failure (HF), and its prevalence is rising. Based on the American Heart Association's Statistical Update in 2021, HF prevalence is predicted to include 6 million people, which is approximately 1.8% of the total US population [11]. Diabetes mellitus is regarded as a significant risk factor for the development and progression of HF [12]. IR has a key role in the progression of diabetes mellitus [3]. Furthermore, longitudinal cohorts have shown that independent of known risk factors, such as diabetes, IR can forecast the occurrence of HF among the general population [13–15].

Given this background, we targeted the general adult American population from 2009 to 2018 to evaluate the prevalence trend of IR, which was detected through the TyG index and HOM-IR, and explore the association of the TyG index with the prevalence of HF.

## Methods

### Data source and study population

**Fig. 1:**
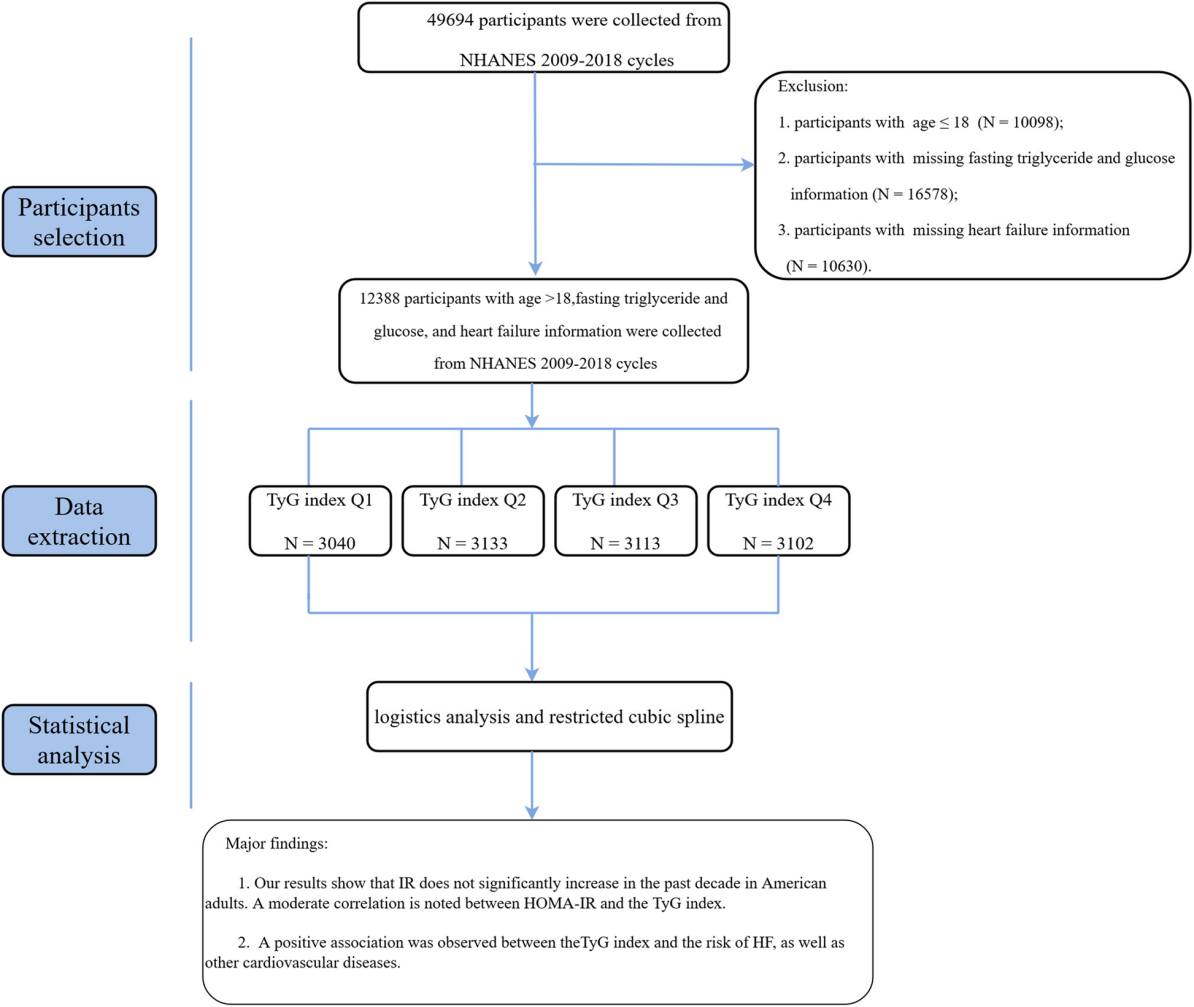
Study selection from the National Health and Nutrition Examination Survey 2009–2018, workflow, and major findings of the present study. Abbreviations: NHANES: National Health and Nutrition Examination Survey; IR: insulin resistance; TyG index: triglyceride-glucose index; HOMA-IR: homeostatic model assessment of insulin resistance

The research is a cross-sectional study that used data from the Nation Health and Nutrition Examination Survey (NHANES) (2009–2018). The whole study workflow is exhibited in Fig. 1. The NHANES is a nationwide representative cross-sectional survey with a complex survey design that is conducted in the United States each year, involving approximately 5000 nationally representative people. The weighted analysis was applied to explain the complex sampling design of NHANES. We included participants > 18 years old who took part in NHANES 2009–2010, 2011–2012,2013–2014,2015–2016, and 2017–2018 cycle. Besides, subjects with missing data on fasting glucose, triglycerides, and HF were precluded from this study (*N* = 37,306). The study included 12,388 participants from 2009 to 2018. More information on the sampling and exclusion criteria is shown in Fig. 1.

### Data collection

The NHANES is conducted every 2-year, and the survey consists of interviews and physical examinations. Participants need to complete interviews, undergo physical examinations, and provide blood samples. The data about age, race and ethnicity, gender, education, marital status and economic status, and medical history was collected by a self-reported, standardized questionnaire [16].

Weight in kilograms divided by height in meters squared is used to compute the body mass index (BMI). After more than five minutes of rest, Systolic blood pressure (SBP) and diastolic blood pressure (DBP) was obtained by trained personnel using a mercury sphygmomanometer, and the average of three readings was recorded (in mmHg). Based on responses to surveys asking participants if they currently smoked cigarettes, smoking status was determined. Based on responses to surveys asking participants if they were drinking right now, drinking status was determined. Physical activity (PA) was determined according to answers to a survey about whether a subject was vigorously or moderately active in recreational activities and how much time they usually spend sitting every day.

Blood samples were taken at the mobile exam facility and stored at 20 °C before being sent to the central labs, where they used standard methods to measure total cholesterol (TC), high-density lipoprotein cholesterol (HDL), uric acid (UA), and creatinine (CR). For evaluating the fasting triglycerides, low-density lipoprotein cholesterol (LDL-C), glucose, and insulin were used 8-h fasting blood specimens were only obtained from a subset of survey subjects.

Coronary heart disease (CHD), stroke **(**cerebrovascular disease**)**, and chronic kidney disease (CKD) were defined as previously diagnosed as CHD, stroke (cerebrovascular disease), and CKD, respectively. Diabetes mellitus was diagnosed based on fasting glucose levels ≥ 7.0 mmol/l [17], or the use of hypoglycemic medications, or a history of diabetes. Systolic blood pressure ≥ 140 mm Hg and/or diastolic blood pressure ≥ 90 mm Hg and use of antihypertensive medications, or a history of hypertension were used to diagnose hypertension. TC levels ≥ 240 mg/dL (6.2 mmol/L) [11], or the use of lipid-lowering drugs, or a history of dyslipidemia were all considered to be diagnostic criteria for dyslipidemia. The estimated glomerular filtration rate (eGFR) was determined using the algorithm used by the Chronic Kidney Disease Epidemiology Collaboration (CKD-EPI) [18].

### Definitions of triglyceride glucose index and homeostatic model assessment of insulin resistance

The TyG index is calculated using the equation Ln [fasting triglycerides (mg/dL) × fasting glucose (mg/dL)/2] [6]. The HOMA-IR was calculated using the formula fasting insulin (microU/L) x fasting glucose (nmol/L)/22.5 [19]. According to the previous study, the cut-off points of HOMA-IR for estimating the prevalence of IR were 2.0 and more than 75% HOMA-IR, respectively [20]. For evaluating the fasting triglycerides, LDL-C, glucose, and insulin were used 8-h fasting blood specimens were only obtained from a subset of survey subjects.

### Ascertain of heart failure

Participants were assessed as having congestive heart failure due to one of the following reasons: if they answered “yes” to the question “Has a doctor or other health professional ever told you that you had congestive HF?”.

### Covariate

The selected covariates consisted of age, gender, marital status (married, never married, other) race (Mexican American, other Hispanic, Non-Hispanic White, Non-Hispanic Black, other Race), education (primary school graduate or below, middle/high/special school, and college graduate or above), current smoking (yes or no), family monthly poverty level category (low, moderate, high), PA (moderate, vigorous), sedentary, BMI, LDL-C, eGFR, UA, the chronic disease (diabetes mellitus, hypertension, stroke, CKD, dyslipidemia).

### Statistical analysis

Continuous variables were exhibited in the form of weighted means and standard error (SE), and categorical variables were reported as weighted frequency percentages [21]. The quartiles are used to convert the TyG index into categorical variables. The TyG index quartiles are Q1 (8.12), Q2 (8.12< 8.55), Q3 (8.55< 9.0), and Q4 (≥ 9.0). The Chi-squared test or Kruskal–Wallis H test was employed to compare the differences in population characteristics by TyG index quartiles.

The correlation between the HOMA-IR and the TyG index was evaluated using the Pearson correlation coefficient. Participants were divided into TyG quartiles. Binary weighted logistics regression analysis was used to evaluate the association between TyG index levels and HF, and the outcome was presented as odds ratios (ORs) and 95% confidence intervals (CIs) with three pre-defined models. Model I was adjusted based on age, sex, and race; model II was adjusted based on Model I, marital status, BMI, education status, LDL-C, eGFR, UA, diabetes mellitus, hypertension, and dyslipidemia; model III was adjusted based on Model II, stroke, CKD, family monthly poverty level category, moderate PA, sedentary, current smoking. Moreover, the restricted cubic spline curves were applied to evaluate the non-linear association between the TyG index and HF.

The sensitivity analyses were used to assess whether the results were stable. We assessed whether the employment of indicator variables for missing data (BMI, LDL-C, eGFR, UA, hypertension, and dyslipidemia) leads to bias by conducting a multiple-imputation analysis which is based on 5 replications and the Markov-chain Monte Carlo method [22]. We also performed sensitivity analyses using a complete-case analysis.

We performed the subgroup analyses for further exploration, the possible impact changes of the relationship between TyG index levels and HF were evaluated for the following factors: sex, age (< 60 vs ≥ 60 years), BMI (< 30 vs ≥ 30 kg/ m^2^), current smoking (yes vs no), eGFR (< 60 vs ≥ 60 ml/min/1.73m^2^), hypertension (yes vs no), diabetes mellitus (yes vs no), and stroke (yes vs no), dyslipidemia (yes vs no), family monthly poverty level category (low vs moderate, low vs high). The subgroup analysis was compared with the highest quartile and quartiles 1–3 to increase the statistic power. A two-sided *p*-value of < 0.05 was considered statistically significant in all analyses. All data analyses were performed using R software version 4.1.3 (www.R-project.org) and Empower version 2.2.0 (www.empowerstats.com, X&Y Solutions, Inc).

## Results

### Baseline characteristics of study participants

**Fig. 2:**
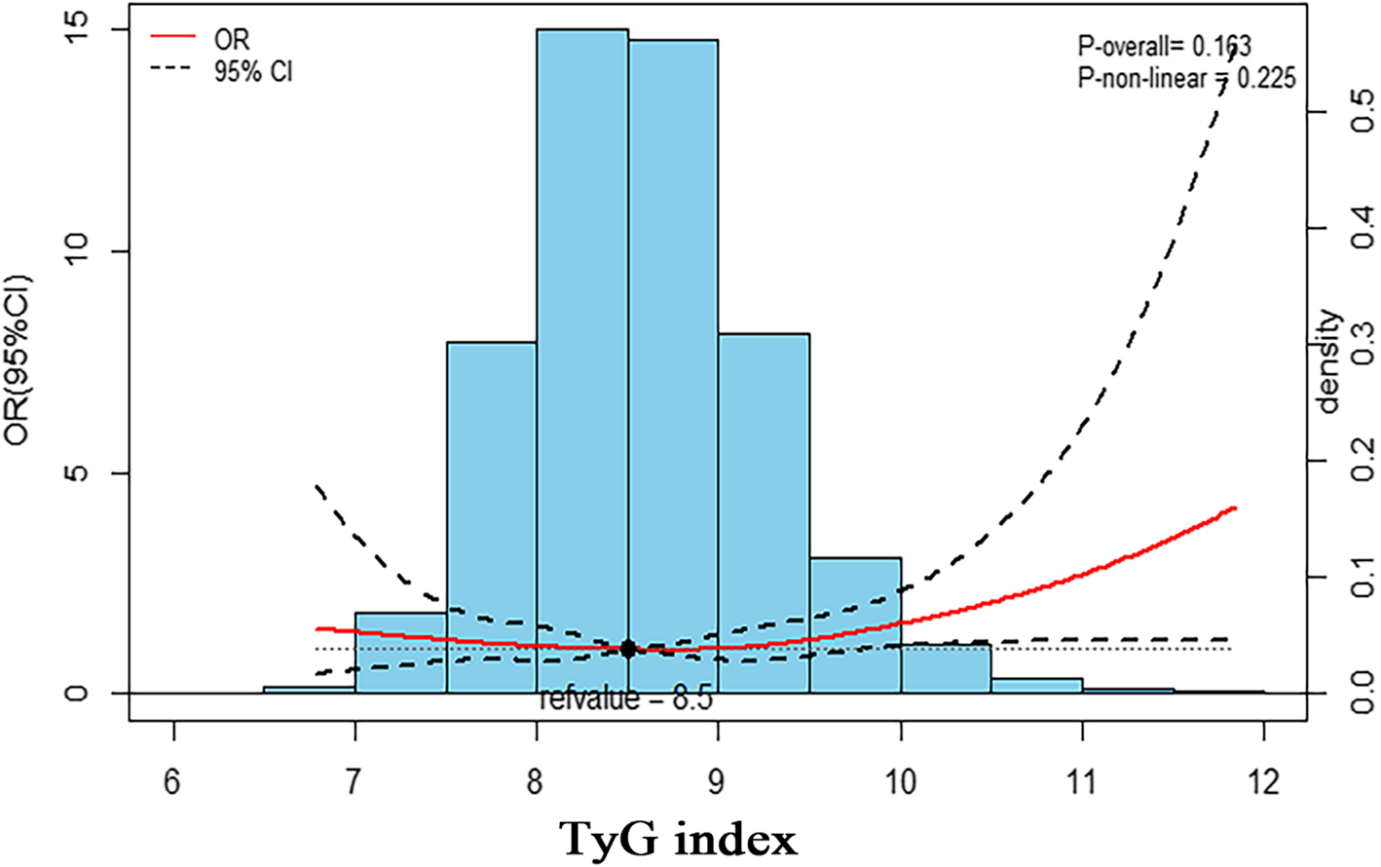
The odds ratio and the histogram of the probability distribution for heart failure according to the triglyceride-glucose index. The red curve with a light black dotted line indicates the adjusted OR with a 95% CI for HF according to the TyG index with a reference of 8.5. The number of knots for the cubic spline curves was three in the model. Adjustment factors included age, sex, race, marital, BMI, education status, LDL-C, eGFR, UA, diabetes mellitus, hypertension, stroke, CKD, dyslipidemia, family monthly poverty level category, moderate PA, sedentary, and current smoking. Abbreviations: OR: odds ratio; CI: confidence interval; HF: heart failure; TyG: triglyceride-glucose index; BMI: body mass index; LDL-C: low-density lipoprotein cholesterol; UA: uric acid; CKD: chronic kidney dysfunction; eGFR: estimated glomerular filtration rate; PA: physical activity

As Fig. 1 reveals, after excluding 37,306 patients with age ≤ 18 years old and missing data on exposure variables (fasting triglycerides and glucose) and HF. The present study has 12,388 participants. The distribution of the TyG index is displayed in Fig. 2. Baseline characteristics for study subjects stratified by the TyG index quartiles are exhibited in Table 1. Generally, the average (SE) age was 47.5 (0.3) years old. Most participants were non-Hispanic whites (65.3%), and 48.2% were males. Participants with higher TyG index levels were more likely to be males, older, smokers, Mexican American, married, poor economically, with lower education status, and with lower PA. Meanwhile, they have higher BMI, SBP, DBP, TC, LDL-C, eGFR, and UA, and lower HDL-C. And they have higher chronic diseases, including HF, hypertension, diabetes mellitus, CHD, stroke, CKD, and dyslipidemia (all *p* < 0.05).

**Table 1:** Weighted baseline characteristics by the quartile of the triglyceride-glucose index of adult Americans from the Nation health and nutrition examination survey 2009–2018

**Characteristics**	**Total**	**Quartiles of triglyceride-glucose index**				P
		Q1 (< 8.12) 3040 (24.5%)	Q2 (8.12- 8.55) 3133 (25.3%)	Q3 (8.55- 9.0) 3113 (25.2%)	Q4 (≥ 9.0) 3102 (25.0%)	
	12,388					
TyG index	8.6 (0)	7.8 (0)	8.3 (0)	8.8 (0)	9.5 (0)	< 0.01
Age, year	47.5 (0.3)	41.8 (0.5)	47.6 (0.5)	48.4 (0.5)	52.1(0.4)	< 0.01
Female, n%	6417 (51.8)	1885 (62.0)	1660 (53.0)	1494 (48.0)	1378 (44.4)	< 0.01
BMI, kg/m^2^	29.2 (0.1)	26.4 (0.2)	28.5 (0.2)	30.3 (0.2)	31.9 (0.2)	< 0.01
DBP, mm Hg	70 (0.2)	68 (0.3)	70 (0.3)	71 (0.4)	72 (0.4)	< 0.01
SBP, mm Hg	122 (0.3)	116 (0.4)	121 (0.4)	123 (0.4)	127 (0.5)	< 0.01
**Smoking status, n (%)**						< 0.01
Never smoke	6962 (56.2)	1930 (63.5)	1831 (58.4)	1681 (54.0)	1520 (49.0)	
Former smoke	5438 (43.9)	1113 (36.6)	1305 (41.7)	1435 (46.1)	1585 (51.1)	
Current smoking	2601 (21.0)	505 (16.6)	636 (20.3)	685 (22.0)	775 (25.0)	
**Current alcohol drinking, n (%)**	2577 (20.8)	517 (17.0)	624 (19.9)	698 (22.4)	738 (23.8)	< 0.01
**Race (%)**						< 0.01
Mexican American	1078 (8.7)	186 (6.1)	241 (7.7)	296 (9.5)	355 (11.4)	
Other Hispanic	768 (6.2)	180 (5.9)	194 (6.2)	199 (6.4)	195 (6.3)	
Non-Hispanic White	8089 (65.3)	1845 (60.6)	2068 (66.0)	2067 (66.4)	2109 (68.0)	
Non-Hispanic Black	1400 (11.3)	556 (18.3)	385 (12.3)	264 (8.5)	195 (6.3)	
Other Race	1053 (8.5)	274 (9.0)	244 (7.8)	286 (9.2)	249 (8.0)	
**Marital status, n (%)**						< 0.01
Never married	2279 (18.4)	781 (25.7)	589 (18.8)	507 (16.3)	402 (13.0)	
Married	6801 (54.)	1520 (50)	1720 (54.9)	1734 (55.7)	1827 (58.9)	
Other	3308 (26.7)	739 (24.3)	824 (26.3)	872 (28.0)	873 (28.1)	
**Education status, n (%)**						< 0.01
Primary school graduate or below	3047 (24.6)	544 (17.9)	745 (23.8)	859 (27.6)	899 (29)	
Middle/high/special school	6169 (49.8)	1532 (50.4)	1632 (52.1)	1516 (48.7)	1489 (48)	
College graduate or above	3171 (25.6)	964 (31.7)	756 (24.1)	738 (23.7)	713 (23)	
**Family monthly poverty level category, n (%)**						< 0.01
Low	3370 (27.2)	775 (25.5)	830 (26.5)	896 (28.8)	869 (28.0)	
Moderate	1549 (12.5)	359 (11.8)	401 (12.8)	386 (12.4)	403 (13.0)	
High	7470 (60.3)	1906 (62.7)	1890 (60.7)	1830 (58.8)	1830 (59.0)	
**Physical activity, n (%)**						< 0.01
Moderate	5637 (45.5)	1623 (53.4)	1416 (45.2)	1363 (43.8)	1235 (39.8)	
Vigorous	3122 (25.2)	1149 (37.8)	843 (26.9)	599 (19.2)	531 (17.1)	
Sedentary/min	393.7 (5.1)	377.7 (7.8)	404.7 (14.6)	392.5 (5.3)	400.8 (5.9)	0.07
**Laboratory results**						
TC, mmol/L	4.9 (0.0)	4.5 (0.0)	4.9 (0.0)	5.1 (0.0)	5.4 (0.0)	< 0.01
Triglycerides, mg/dL	120.4 (1.4)	52.5 (0.4)	84.7 (0.3)	122.7 (0.6)	231.6 (3.6)	< 0.01
HDL-C, mmol/L	1.4 (0.0)	1.7 (0.0)	1.5 (0.0)	1.3 (0.0)	1.1 (0.0)	< 0.01
LDL-C, mg/dL	113.3 (0.5)	99.5 (0.8)	113.6 (0.7)	120.8 (0.8)	120.6 (0.9)	< 0.01
Fasting glucose, mg/dL	106.8 (0.4)	94.7 (0.3)	100.5 (0.3)	105.3 (0.5)	128.8 (1.2)	< 0.01
BUN mmol/L	4.9 (0)	4.7 (0)	4.9 (0)	4.9 (0)	5.2 (0)	< 0.01
Cr mmol/L	77.5 (0.4)	74.0 (0.6)	77.6 (0.7)	78.2 (0.7)	80.4 (0.8)	< 0.01
eGFR ml/min/1.73m^2^	120.5 (0.8)	116.0 (1.3)	117.2 (1.2)	123.3 (1.4)	126.1 (1.4)	< 0.01
UA, umol/L	324.8 (1.1)	291.0 (1.9)	317.5 (1.9)	337.3 (2.3)	357.2 (2.1)	< 0.01
HbA1c	5.4 (0)	5.3 (0.0)	5.5 (0.0)	5.6 (0.0)	6.3 (0.0)	< 0.01
HOMA-IR	1.3 (0)	0.9 (0.0)	1.1 (0.0)	1.2 (0.0)	2.0 (0.1)	< 0.01
**Drugs**						
Lipid-lowering drugs	4315 (34.8)	565 (18.6)	958 (30.8)	1170 (37.6)	1622 (52.3)	< 0.01
Hypoglycemic agents	1228 (9.9)	67 (2.2)	140 (4.5)	274 (8.8)	747 (24.1)	< 0.01
**History of disease, n (%)**						
Hypertension	5253 (42.4)	942 (31.0)	1300 (41.5)	1466 (47.1)	1545 (49.8)	< 0.01
Diabetes mellitus	2317 (18.7)	316 (10.4)	473 (15.1)	694 (22.3)	834 (26.9)	< 0.01
CHD	446 (3.6)	58 (1.9)	94 (3.0)	121 (3.9)	173 (5.6)	< 0.01
Stroke	359 (2.9)	58 (1.9)	89 (2.8)	97 (3.1)	115 (3.7)	< 0.01
CKD	322 (2.6)	52 (1.7)	72 (2.3)	81 (2.6)	117 (3.8)	< 0.01
Dyslipidemia	5760 (46.5)	857 (28.2)	1319 (42.1)	1675 (53.8)	1909 (61.5)	< 0.01
HF	322 (2.6)	40 (1.3)	63 (2.0)	85 (2.7)	136 (4.4)	< 0.01

Data are expressed as mean (SE) and numbers (percentage) as appropriate. All estimates were weighted to be nationally representative

Abbreviations: *SE* Standard error, *TyG index* Triglyceride-glucose index, *BMI* Body mass index, *DBP* Diastolic blood pressure, *TC* Total cholesterol, *HDL-C* High-density lipoprotein cholesterol, *LDL-C* Low-density lipoprotein cholesterol, *SBP* Systolic blood pressure, *UA* Uric acid, *HbA1c* Glycated hemoglobin, *BUN* Urea nitrogen, *Cr* Creatinine, *eGFR* Estimated glomerular filtration rate, *HOMA-IR* Homeostatic model assessment of insulin resistance, *CHD* Coronary heart disease, *CKD* Chronic kidney dysfunction, *HF* Heart failure

### The prevalence values of Insulin resistance

**Fig. 3:**
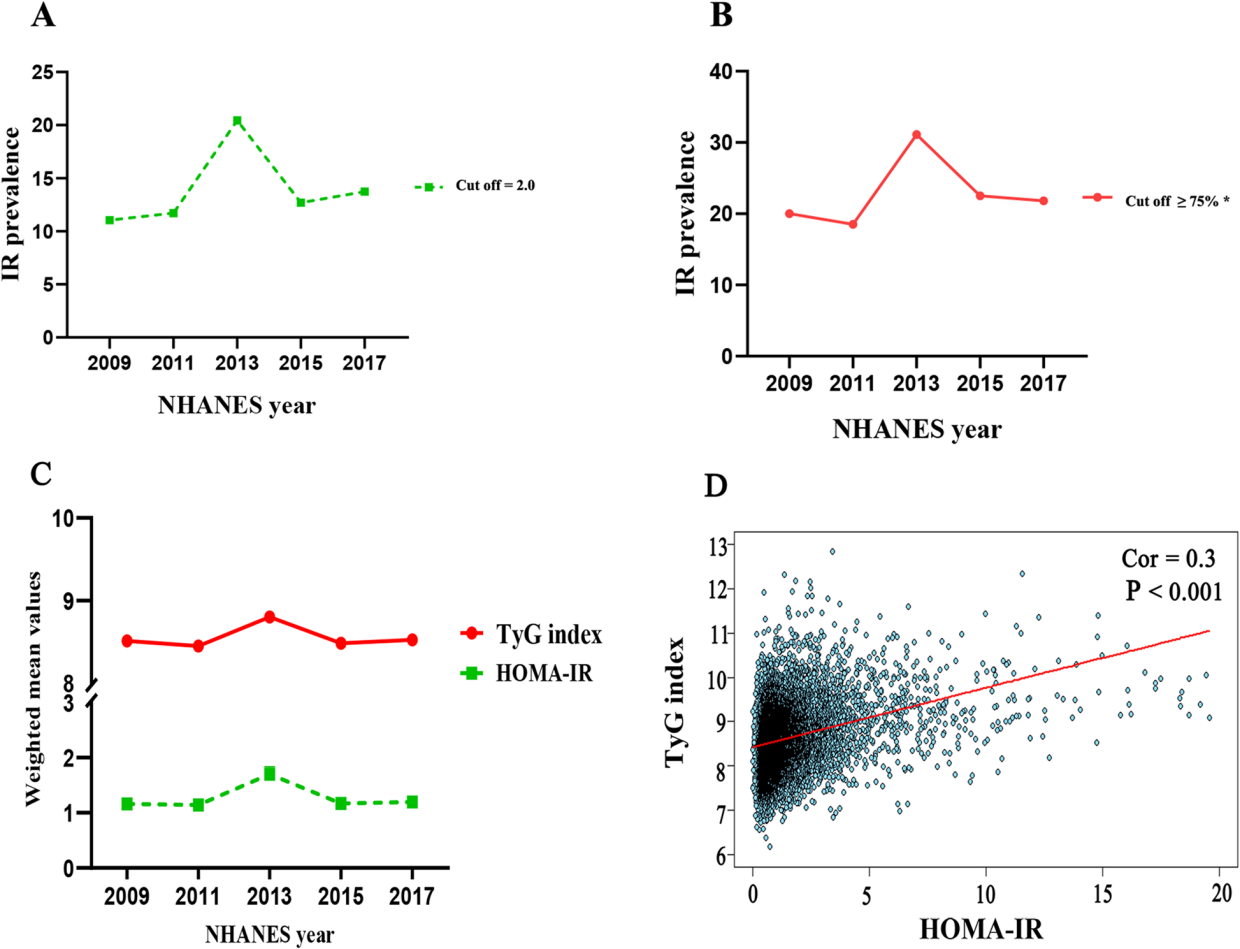
The prevalence of insulin resistance, and the changing trend of homeostatic model assessment of insulin resistance and triglyceride-glucose index in adult Americans from the Nation Health and Nutrition Examination Survey 2009–2018. **A**-**B**: The prevalence of IR was defined as having a HOMA-IR greater than 2.0 (A) or 1.5 (B). **C**: An overview of the five NHANES cycles (2009–2018) that show the changing trend of the TyG index and HOMA-IR mean values. **D**: The correlation between the TyG index and HOMA-IR. Abbreviations: IR: insulin resistance; TyG index: triglyceride-glucose index; HOMA-IR: homeostatic model assessment of insulin resistance

From 2009 to 2018, the mean prevalence of IR was 1.3. The highest prevalence of IR was observed in 2013–2014, with 20.45% (cut off 2.0) and 31.3% (cut off 1.5) (Fig. 3 A-B), with a similar trend in terms of the TyG index and HOMA-IR(Fig. 3 C). HOMA-IR and the TyG index showed a moderately positive linear correlation (*r* = 0.30, *P* < 0.01). (Fig. 3 D). In addition, we investigated the trend of values of the TyG index stratified by demographic factors. As presented in Figure S1, the TyG index increased with age (Figure S1 A). Males had a higher TyG index than females (Figure S1 B). Regarding race, the TyG index level of Mexican Americans was greater than that of other races (Figure S1 C). Similar results of HOMA-IR were noted when patients were stratified by age, sex, and race.

### Triglyceride glucose index stratified by demographic-related factors

As Figure S2 shows, men exhibited a 1.76-times (95% CI: 1.53–2.01, *P* < 0.001) greater TyG index values than women. Compared with low education status, moderate education status, and high education status had a lower TyG index values of 0.79- times (95% CI: 0.69–0.90, *P* < 0.01) and a 0.67- times (95% CI: 0.56–0.79, *P* < 0.01), respectively. Compared to Mexican Americans, other Hispanics, non-Hispanic whites, non-Hispanic blacks, and other races had a lower TyG index of 0.70- times (95% CI: 0.57–0.85, *P* < 0.01), 0.62- times (95% CI: 0.54–0.70, *P* < 0.01), 0.25- times (95% CI: 0.21–0.30, *P* < 0.01) and 0.75- times (95% CI: 0.61–0.91, *P* < 0.01), respectively. Vigorous PA had a 0.71-fold (95% CI: 0.61–0.83, *P* < 0.001) lower TyG index than non-vigorous PA. Moderate PA had a 0.82- times (95% CI: 0.71–0.95, *P* < 0.001) lower TyG index than non-moderate PA. Current smoking was associated with a 1.31-fold (95% CI: 1.15–1.50, *P* < 0.001) higher TyG index than noncurrent smoking. Current drinking exhibited a 1.65-fold (95% CI: 1.36–2.02, *P* < 0.001) greater TyG index than non-current drinking.

### Associations between the triglyceride glucose index and heart failure

HF prevalence was 2.6% (322/12388) among adults in the U.S. from 2009 to 2018. The TyG index was appraised as a categorical variable. Compared to participants in quartile 1 of the TyG index, those in quartiles 2, 3, and 4 had weighted multivariate-adjusted ORs of 0.91 (95% CI: 0.52–1.56), 1.13 (95% CI: 0.71–1.80), and 1.45 (95% CI: 0.87–2.41), respectively. After adjustment for age, sex, race, marital, BMI, education status, LDL-C, eGFR, UA, diabetes mellitus, hypertension, stroke, CKD, dyslipidemia, family monthly poverty level category, moderate PA, sedentary, and current smoking. The continuous variables analysis showed a positive association (per 1 unit increase; OR: 1.34; 95% CI: 1.02–1.76) (Table 2). As presented in Fig. 2, the relationship between the TyG index and HF seemed to be linear (P-non-linear = 0.23) after adjustments. Supplemental analysis revealed that subjects in quartile 4 of the TyG index (≥ 9) had a significantly higher prevalence of HF (OR: 1.41; 95% CI: 1.01–1.95) than those in quartiles 1–3 of the TyG index (< 9).

**Table 2:** Association of the triglyceride-glucose index with a prevalence of Heart failure in adult Americans from the Nation Health and Nutrition Examination Survey 2009–2018

TyG index	Cases/N	Model I OR (95%CI)	*P* value	Model II OR (95%CI)	*P*-value	Model III OR (95%CI)	*P*-value
Per 1 unit increase	342/12388	1.76 (1.37,2.27),	< 0.001	1.40 (1.07,1.83),	0.02	1.34 (1.02,1.76)	0.04
Quartiles							
Q1 (< 8.12)	61/3040	Ref	1.0	Ref	1.0	Ref	1.0
Q2 (8.12- 8.55)	94 /3133	1.17 (0.72,1.91)	0.53	1.04 (0.61,1.76)	0.89	0.91 (0.52,1.56)	0.73
Q3 (8.55- 9.0)	93/3113	1.53 (0.99,2.36)	0.06	1.21 (0.78,1.87)	0.40	1.13 (0.71,1.80)	0.59
Q4 (≥ 9.0)	155/3102	2.41 (1.45,4.01)	< 0.01	1.55 (0.96,2.50)	0.08	1.45 (0.87,2.41)	0.16
P for trend		< 0.001		< 0.001		< 0.001	
Categories							
Q1-Q3 (< 9.0))	279/9286	Ref	1.0	Ref	1.0	Ref	1.0
Q4 (≥ 9.0)	155/3102	1.91 (1.39,2.62)	< 0.001	1.40 (1.02,1.90)	0.04	1.41 (1.01,1.95),	0.05

Model I was adjusted for age, gender, and race; Model II was adjusted for Model I, marital status, BMI, education status, LDL-C, eGFR, UA, diabetes mellitus, hypertension, dyslipidemia; Model III was adjusted for Model II, stroke, CKD, Family monthly poverty level category, moderate PA, sedentary, current smoking

Abbreviations: *95% CI* 95% confidence interval, *HF* Heart failure, *TyG index* Triglyceride-glucose index, *OR* Odds ratio, *BMI* Body mass index, *LDL-C* Low-density lipoprotein cholesterol, *UA* Uric acid, *eGFR* Estimated glomerular filtration rate, *CKD* Chronic kidney dysfunction, *PA* Physical activity

### Association of the triglyceride-glucose index with other diseases

We found that the TyG index was remarkably associated with the increased prevalence of dyslipidemia (OR: 2.55; 95% CI: 2.31–2.81), CHD (OR: 1.41; 95% CI: 1.07–1.83), and hypertension (OR: 1.56; 95% CI: 1.43–1.70), after adjustment for age, sex, race, BMI, current smoking, marital status, Family monthly poverty level category, and education status. But the TyG index was not significantly associated with the increased prevalence of stroke **(**cerebrovascular disease**)** (OR: 1.07; 95% CI: 0.86–1.34). (Table S1).

### Sensitivity analyses and subgroup analyses

The multiple imputation analysis generated consistent results (OR, 1.4; 95% CI, 1.1–1.7), suggesting that missing variables did not cause bias (Table S2).

**Fig. 4:**
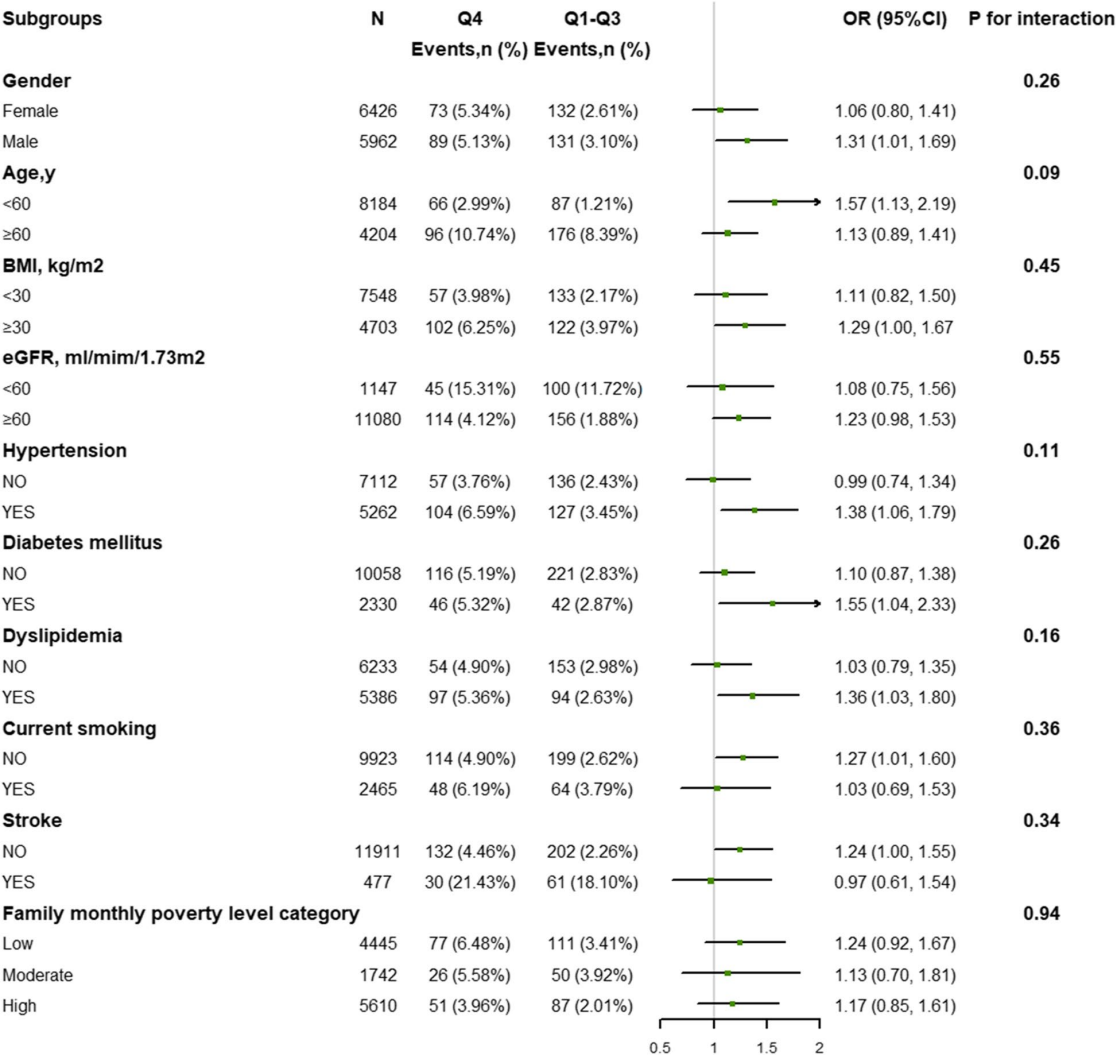
The association between the triglyceride-glucose index (Q4 vs. Q1-Q3) and heart failure in various subgroups after adjustments. The adjusted confounders were age, sex, race, marital status, BMI, education status, LDL-C, eGFR, UA, diabetes mellitus, hypertension, dyslipidemia, stroke, CKD, family monthly poverty level category, moderate PA, sedentary, current smoking (confounders were not adjusted if they were listed as a stratified factor in subgroups). Abbreviations: HF: heart failure; BMI: body mass index; TyG index: triglyceride-glucose index; LDL-C: low-density lipoprotein cholesterol; CKD: chronic kidney dysfunction; UA: uric acid; eGFR: estimated glomerular filtration rate; PA: physical activity

Subgroup analyses were used to assess the association between the TyG index and HF in per-defined subgroups (Fig. 4). No significant interactions between the TyG index and sex, age, BMI, eGFR, hypertension, diabetes mellitus, stroke, current smoking, family monthly poverty level category, or dyslipidemia were noted (all p interactions > 0.1). In addition, we used a propensity score matching to strengthen our findings (OR = 1.52, 95%CI: 1.17, 1.98) (Table S3-S4). Meanwhile, sensitivity analysis additionally by adjusting hypoglycemic agents and lipid-lowering drugs showed the result was consistent with the main results (Table S5).

## Discussion

### Major findings

The study is a large-scale cross-sectional study of US adults**,** our results showed that the IR prevalence estimated via the TyG index and HOMA-IR was stable in Americans from 2009 to 2018, but the prevalence was relatively higher from 2013–2014. A moderate positive correlation was observed between HOMA-IR and the TyG index. Males, low education, other races, lower PA, smoking, and drinking were all associated with a higher TyG index. Finally, our study found that the TyG index was associated with an increased risk of HF as well as other cardiovascular diseases.

As a simple and new marker, the TyG index was applied to assess the severity of IR among patients with diabetes mellitus [3]. A previous systematic review explored the risk of CVDs associated with the TyG index. However, it is primarily based on the East Asian population [9]. For example, a study from China illustrated a significantly positive association between the TyG index and adverse cardiovascular outcomes [23]. Another prospective study from Korea indicated that the TyG index was considered an independent factor in the prediction of the development of coronary artery calcification [24]. A study showed that the TyG index predicts adverse cardiovascular events among Chinese patients with diabetes and acute coronary syndrome independently of recognized cardiovascular risk factors [25]. In another cross-sectional survey that enrolled the Chinese elderly population, there is a significant association between the TyG index and hypertension, and compared to lipid and glycemic indices, the TyG index shows higher discriminative power for hypertension [26]. However, the evidence for TyG and associated cardiovascular risk based on general US adults is limited [27]. Based on a well-designed national survey, our study first explored the prevalence and associated HF and cardiovascular events with TyG in adult Americans.

Some studies show that the TyG index is moderately positively correlated with HOMA-IR [28, 29]. However, we found the correlation between the TyG index and HOMA-IR is lower and may be explained by the following reason. The main purpose of the study is to investigate the association between the TyG index and the prevalence of HF. Therefore, in the subject selection process, the individuals with missing HF information were excluded. Meanwhile, the population with diabetes or hyperlipidemia has been treated with lipid-lowering and hypoglycemic drugs, and the blood sugar and lipid levels are affected. Thus, there may be a reduced correlation between the TyG index and HOMA-IR.

From 2009 to 2018, diabetes prevalence in America has increased [30]. However, our study found that the prevalence of IR was stable in US adults. Several potential reasons may explain this inconsistency. First, to investigate the risk of HF associated with the TyG index, subjects with missing information on HF and the TyG index were deleted, which may reduce the representation of individuals from the US. Second, the population with type 2 diabetes was a relatively small proportion of the overall population in the present study (18.7%), which diluted the effect of IR reflecting the type 2 diabetes prevalence. Third, diabetes has two main forms, including type 1 and type 2, and IR has traditionally been related to type 2 diabetes. Type 2 diabetes is remarkably heterogeneous [31]. In some subgroups of patients with type 2 diabetes at the early stage, IR has not occurred or is not evident. Therefore, the prevalence of IR may not reflect diabetes prevalence. In a study of the diabetes prevalence based on race and ethnicity in the US, the results indicated that diabetes prevalence varies by race and ethnicity, and Mexican Americans exhibited the highest prevalence of total diabetes [32]. Our results found that the TyG index was the highest among Mexican Americans. Variations in the diabetes prevalence between races/ethnicities could be due to physiological differences [32].

IR has an important pathophysiologic role in type 2 diabetes, and IR is frequently prevalent in HF patients [33]. In addition, a study also demonstrated that IR was an indicator of deterioration in heart function [34]. A meta-analysis showed the association between IR and the risk of HF developing after adjusting for traditional risk factors (RR: 1.08; 95% CI: 1.04–1.11). This association has persisted in studies involving individuals with and without diabetes [35]. Our results further reinforced these studies.

Obesity has been identified as a major risk factor for HF. According to the Framingham Heart Study, increases in BMI of 1 kg/m^2^ increased the risk of HF by 5% in men and 7% in women [36]. This finding may emphasize the necessity of a weight-specific control measure to prevent HF. In the subgroup analysis, we found no significant association between the TyG index and HF in the population with BMI < 30 kg/m^2^ (OR = 1.20, 95% CI: 0.88–1.63). However, the interaction was not significant for obesity. The findings demonstrated that the TyG index is a risk factor for HF independent of obesity. Another vital effect modifier is diabetes mellitus. A meta-analysis reported the adverse cardiovascular events among coronary artery disease patients associated with the TyG index, and the risk between TyG and the primary outcome (including HF) was consistent in patients with and without diabetes mellitus (with diabetes mellitus: HR = 2.49, 95% CI: 1.86–3.34; without diabetes mellitus: HR = 1.95, 95% CI: 1.20–3.17) [37]. Our study included 10,058 people who did not have diabetes mellitus and 2,330 diabetes mellitus patients. The subgroup results indicated the association between a high TyG index and HF in diabetes mellitus (OR = 1.55, 95% CI: 1.02–2.37). In the normal population, the association became nonsignificant (OR = 1.19, 95% CI: 0.95–1.50). Although a nonsignificant interaction was found, more studies are needed to ascertain the interaction between diabetes mellitus and the TyG index.

Our results showed the association between the TyG index and the prevalence of CHD, diabetes mellitus, hypertension, dyslipidemia, and CKD. These results were compatible with another research [4, 6, 7]. However, the TyG index was not significantly associated with the increased risk of stroke. Our result is not consistent with existing research. Zhao et al. showed that in the general population, the TyG index is an independent predictor of ischemic stroke [38]. Zhao et al. conducted a prospective cohort study in rural China, enrolling only people over the age of 40. However, our study enrolled US adults exhibiting a greater age range. This feature may explain why our results are not consistent with those of Zhao et al. [38].

### Comparisons with previous studies

Our finding is in line with earlier studies. There is a significant association between the TyG index and an increased prevalence of CVD incidence, which was more outstanding among the young people [39]. Our results showed that the TyG index is significantly associated with an increased risk of HF in the younger population (age < 60 years), although insignificant interactions are noted between the TyG index and age.

Guo et al. demonstrated the adverse events in HF patients with type 2 diabetes associated with the TyG index. Compared with the lowest TyG index group, the highest TyG index group has a higher risk of adverse events in HF (HR: 2.15, 95% CI: 1.37–3.35) [40]. Yang et al. revealed that a higher TyG index is associated with all-cause death or HF rehospitalization (HR = 2.01, 95% CI: 1.03–4.01) [41]. Our study first reported i) ten-year data on IR epidemiological trends revealed a moderately positive correlation between HOMA-IR and the TyG index in general US adults; and ii) the TyG index and the prevalence of HF were positively associated, as were other cardiovascular diseases.

### Underlying mechanism

The association between the TyG index and increased risk of HF may be explained as follows: First, maintaining myocardial function is dependent on glucose uptake and metabolism, and the bioavailability of glucose is limited by IR, leading to myocardial energy acquisition from fatty acid metabolism [42]. These changes result in fatty acid accumulation, which causes lipotoxicity and worsens HF through mitochondrial dysfunction and apoptosis [43]. Second, IR is associated with chronic inflammation, arterial stiffness and coronary artery calcification, which prompts cardiomyocyte injury, and fibrosis, impairing both diastolic and systolic functions [44]. Third, IR has been demonstrated to stimulate the sympathetic nervous system [45] and the renin–angiotensin–aldosterone system [46]. Both above systems are associated with myocardial fibrosis and cardiac dysfunction [44, 47]. Fourth, calcium has a key role in regulating mitochondrial function [48] and cardiac diastolic function [49], but IR can weaken cardiac calcium processing capacity [47] (Figure S3).

### Clinical practices

Our finding has may clinical importance for the prevention of the development of HF in the general population. In practice, HOMA-IR has been the most broadly applied method [3]. There are studies that have demonstrated that the TyG index is superior to the HOMA-IR for predicting IR, type 2 diabetes, and coronary artery stenosis [3, 50, 51]. In our study, the participants with higher TyG index levels were associated with having a higher prevalence of HF. Therefore, the TyG index may be applied to the risk stratification of those with a high risk of HF. The TyG index has a relatively low cost as a marker of HF events in clinical practice. Meanwhile, in subgroup analysis, we found that diabetes has no potential effect modifications of the relationship between TyG index levels and HF. This showed that the TyG index used to evaluate the IR could be considered for routine utilization. As we know, IR is considered an early stage in the development of diabetes, and IR could be reversible by intervention [52]. Some studies have shown that hypoglycemic agents can alleviate the prognosis and ameliorate cardiac remodeling or dysfunction in people who do not have diabetes [53, 54].

### Limitations

Several potential limitations should be mentioned. First, as a cross-sectional study. Thus, this research does not imply causality. Second, HF-related data were gathered from self-reported information, and patients with undiagnosed HF were not included. Meanwhile, because the selection population could have some bias, this may lead to the prevalence of IR being calculated inaccurately. Third, some patients with missing TyG and HF data were excluded from the present study, which largely reduced the sample size from the original cohort. Therefore, all the above two points may cause selection bias. Fourth, we only obtain baseline information about the TyG index. As a result, we were unable to determine whether managing the TyG index might improve participant prognosis. Meanwhile, to eliminate the confounders in the multivariate analysis, we have involved as many clinically relevant variables as possible. However, the potential confounder likelihood remained. Finally, this study was based on American data, and the generalizability of these findings to other regions requires further research.

## Conclusion

This study revealed that the prevelence of IR did not significantly increase from 2009 to 2018 among American. A moderate correlation is noted between HOMA-IR and the TyG index. The present study suggests an association between the TyG index and prevalence of HF among the general American population, and more studies are needed to assess the predictive ablity of TyG index and the HF incidence.

## Supplementary Information

**Additional file 1**: **Table S1.** Association of the triglyceride-glucose index with the prevalence of specific cardiovascular diseases in adult Americans from the Nation Health and Nutrition Examination Survey 2009-2018. **Table S2.** Sensitive analysis of the association between the triglyceride-glucose index and the prevalence of heart failure in adult Americans based on the multiple-imputation analysis. **Table S3.** Baseline characteristics after propensity score matching of adult Americans from the Nation Health and Nutrition Examination Survey 2009-2018. **Table S4.** The associations of the triglyceride-glucose index with the prevalence of heart failure in the propensity score matched from the Nation Health and Nutrition Examination Survey 2008-2018. **Table S5.** The associations of the triglyceride-glucose index with the prevalence of heart failure after adjusted hypoglycemic agents, and lipid-lowering drugs from the Nation Health and Nutrition Examination Survey 2008-2018. **Figure S1.** The weighted mean values of the triglyceride-glucose index in adult Americans from the Nation Health and Nutrition Examination Survey 2009-2018. A: The weighted mean values of the triglyceride-glucose index of different age stages; B: The weighted mean values of the triglyceride-glucose index of a different gender; C: The weighted mean values of the triglyceride-glucose index of different races. Bar graphs and error bars represent weighted mean ± SE. **Figure S2.** Risk factor association with the triglyceride-glucose index. **Figure S3.** The maybe underlying mechanism of insulin resistance-induced heart failure.

## Data Availability

The datasets generated and/or analyzed during the current study are available in the NHANES repository,https://www.cdc.gov/nchs/nhanes/ The datasets used and analyzed during the current study are available from the corresponding author upon reasonable request.

## Abbreviations

TyG index: Triglyceride glucose index
CVD: Cardiovascular disease
HF: Heart failure
NHANES: National health and nutrition examination survey
IR: Insulin resistance
BMI: Body mass index
SBP: Systolic blood pressure
DBP: Diastolic blood pressure
PA: Physical activity
HDL-C: High-density lipoprotein cholesterol
UA: Uric acid
CKD-EPI: Chronic kidney disease epidemiology collaboration
eGFR: Estimated glomerular filtration rate
CR: Creatinine
TC: Total cholesterol
LDL-C: Low-density lipoprotein cholesterol
CHD: Coronary heart disease
CKD: Chronic kidney dysfunction
OR: Odds ratio
CI: Confidence interval
HOMA-IR: Homeostatic model assessment of insulin resistance
SE: Standard error

## References

[1] Bonora E, Formentini G, Calcaterra F, Lombardi S, Marini F, Zenari L, Saggiani F, Poli M, Perbellini S, Raffaelli A (2002). HOMA-estimated insulin resistance is an independent predictor of cardiovascular disease in type 2 diabetic subjects: prospective data from the verona diabetes complications study. Diabetes Care.

[2] Simental-Mendía LE, Rodríguez-Morán M, Guerrero-Romero F (2008). The product of fasting glucose and triglycerides as surrogate for identifying insulin resistance in apparently healthy subjects. Metab Syndr Relat Disord.

[3] Park HM, Lee HS, Lee YJ, Lee JH (2021). The triglyceride-glucose index is a more powerful surrogate marker for predicting the prevalence and incidence of type diabetes mellitus than the homeostatic model assessment of insulin resistance. Diabetes Res Clin Pract.

[4] Jian S, Su-Mei N, Xue C, Jie Z, Xue-Sen W (2017). Association and interaction between triglyceride-glucose index and obesity on risk of hypertension in middle-aged and elderly adults. Clin Exp Hypertens.

[5] Irace C, Carallo C, Scavelli FB, De Franceschi MS, Esposito T, Tripolino C, Gnasso A (2013). Markers of insulin resistance and carotid atherosclerosis. a comparison of the homeostasis model assessment and triglyceride glucose index. Int J Clin Pract.

[6] Hu L, Bao H, Huang X, Zhou W, Wang T, Zhu L, Liu X, Li M, Cheng X (2022). Relationship between the triglyceride glucose index and the risk of first stroke in elderly hypertensive patients. Int J Gen Med.

[7] Sánchez-Íñigo L, Navarro-González D, Fernández-Montero A, Pastrana-Delgado J, Martínez JA (2016). The TyG index may predict the development of cardiovascular events. Eur J Clin Invest.

[8] Lee EY, Yang HK, Lee J, Kang B, Yang Y, Lee SH, Ko SH, Ahn YB, Cha BY, Yoon KH (2016). Triglyceride glucose index, a marker of insulin resistance, is associated with coronary artery stenosis in asymptomatic subjects with type 2 diabetes. Lipids Health Dis.

[9] Liu X, Tan Z, Huang Y, Zhao H, Liu M, Yu P, Ma J, Zhao Y, Zhu W, Wang J (2022). Relationship between the triglyceride-glucose index and risk of cardiovascular diseases and mortality in the general population: a systematic review and meta-analysis. Cardiov Diabetol.

[10] Liu X, Abudukeremu A, Jiang Y, Cao Z, Wu M, Ma J (2023). U-shaped association between the triglyceride–glucose index and atrial fibrillation incidence in a general population without known cardiovascular disease. Cardiov Diabetol.

[11] Virani SS, Alonso A, Aparicio HJ, Benjamin EJ, Bittencourt MS, Callaway CW, Carson AP, Chamberlain AM, Cheng S, Delling FN (2021). Heart disease and stroke statistics-2021 update: a report from the american heart association. Circulation.

[12] Roger VL (2021). Epidemiology of heart failure: a contemporary perspective. Circ Res.

[13] Ingelsson E, Sundström J, Ärnlöv J, Zethelius B, Lind L (2005). Insulin resistance and risk of congestive heart failure. JAMA.

[14] Vardeny O, Gupta DK, Claggett B, Burke S, Shah A, Loehr L, Rasmussen-Torvik L, Selvin E, Chang PP, Aguilar D (2013). Insulin resistance and incident heart failure: the ARIC study (Atherosclerosis Risk in Communities). JACC: Heart Fail.

[15] Banerjee D, Biggs ML, Mercer L, Mukamal K, Kaplan R, Barzilay J, Kuller L, Kizer JR, Djousse L, Tracy R (2013). Insulin resistance and risk of incident heart failure: cardiovascular health study. Circulation: Heart Fail.

[16] Zipf G, Chiappa M, Porter KS, Ostchega Y, Lewis BG, Dostal J (2013). National health and nutrition examination survey: plan and operations, 1999–2010. Vital Health Stat.

[17] Handelsman Y, Bloomgarden ZT, Grunberger G, Umpierrez G, Zimmerman RS, Bailey TS, Blonde L, Bray GA, Cohen AJ, Dagogo-Jack S (2015). American association of clinical endocrinologists and american college of endocrinology - clinical practice guidelines for developing a diabetes mellitus comprehensive care plan - 2015. Endocr Pract.

[18] Cockcroft DW, Gault MH (1976). Prediction of creatinine clearance from serum creatinine. Nephron.

[19] So A, Sakaguchi K, Okada Y, Morita Y, Yamada T, Miura H, Otowa-Suematsu N, Nakamura T, Komada H, Hirota Y (2020). Relation between HOMA-IR and insulin sensitivity index determined by hyperinsulinemic-euglycemic clamp analysis during treatment with a sodium-glucose cotransporter 2 inhibitor. Endocr J.

[20] Sánchez-García A, Rodríguez-Gutiérrez R, Mancillas-Adame L, González-Nava V, Díaz González-Colmenero A, Solis RC, Álvarez-Villalobos NA, González-González JG (2020). Diagnostic accuracy of the triglyceride and glucose index for insulin resistance: a systematic review. Int J Endocrinol.

[21] Cao C, Liu Q, Yang L, Zheng X, Lan P, Koyanagi A, Vancampfort D, Soysal P, Veronese N, Stubbs B (2020). Handgrip strength is associated with suicidal thoughts in men: Cross-sectional analyses from NHANES. Scand J Med Sci Sports.

[22] Park SY, Freedman ND, Haiman CA, Le Marchand L, Wilkens LR, Setiawan VW (2017). Association of coffee consumption with total and cause-specific mortality among nonwhite populations. Ann Intern Med.

[23] Ma X, Dong L, Shao Q, Cheng Y, Lv S, Sun Y, Shen H, Wang Z, Zhou Y, Liu X (2020). Triglyceride glucose index for predicting cardiovascular outcomes after percutaneous coronary intervention in patients with type 2 diabetes mellitus and acute coronary syndrome. Cardiovasc Diabetol.

[24] Won KB, Park EJ, Han D, Lee JH, Choi SY, Chun EJ, Park SH, Han HW, Sung J, Jung HO (2020). Triglyceride glucose index is an independent predictor for the progression of coronary artery calcification in the absence of heavy coronary artery calcification at baseline. Cardiovasc Diabetol.

[25] Akbar MR, Pranata R, Wibowo A, Irvan, Sihite TA, Martha JW. The association between triglyceride-glucose index and major adverse cardiovascular events in patients with acute coronary syndrome - dose-response meta-analysis. Nutr Metab Cardiovasc Dis. 2021;31:3024-3030. 10.1016/j.numecd.2021.08.026 10.1016/j.numecd.2021.08.026 34625361

[26] Zhu B, Wang J, Chen K, Yan W, Wang A, Wang W, Gao Z, Tang X, Yan L, Wan Q (2020). A high triglyceride glucose index is more closely associated with hypertension than lipid or glycemic parameters in elderly individuals: a cross-sectional survey from the reaction study. Cardiovasc Diabetol.

[27] Chen Y, Chang Z, Zhao Y, Liu Y, Fu J, Zhang Y, Liu Y, Fan Z (2021). Association between the triglyceride-glucose index and abdominal aortic calcification in adults: a cross-sectional study. Nutr Metab Cardiovasc Dis.

[28] Lee SB, Kim MK, Kang S, Park K, Kim JH, Baik SJ, Nam JS, Ahn CW, Park JS (2019). Triglyceride glucose index is superior to the homeostasis model assessment of insulin resistance for predicting nonalcoholic fatty liver disease in Korean adults. Endocrinol Metab (Seoul).

[29] Wu TD, Fawzy A, Brigham E, McCormack MC, Rosas I, Villareal DT, Hanania NA (2021). Association of triglyceride-glucose index and lung health: a population-based study. Chest.

[30] Tinajero MG, Malik VS (2021). An update on the epidemiology of type 2 diabetes: a global perspective. Endocrinol Metab Clin North Am.

[31] Pearson ER (2019). Type 2 diabetes: a multifaceted disease. Diabetologia.

[32] Cheng YJ, Kanaya AM, Araneta MRG, Saydah SH, Kahn HS, Gregg EW, Fujimoto WY, Imperatore G (2019). Prevalence of diabetes by race and ethnicity in the United States, 2011–2016. JAMA.

[33] Palmiero G, Cesaro A, Vetrano E, Pafundi PC, Galiero R, Caturano A, Moscarella E, Gragnano F, Salvatore T, Rinaldi L (2021). Impact of SGLT2 inhibitors on heart failure: from pathophysiology to clinical effects. Int J Mol Sci.

[34] Banerjee D, Biggs ML, Mercer L, Mukamal K, Kaplan R, Barzilay J, Kuller L, Kizer JR, Djousse L, Tracy R (2013). Insulin resistance and risk of incident heart failure: cardiovascular health study. Circ Heart Fail.

[35] Erqou S, Adler AI, Challa AA, Fonarow GC, Echouffo-Tcheugui JB (2022). Insulin resistance and incident heart failure: a meta-analysis. Eur J Heart Fail.

[36] Kenchaiah S, Evans JC, Levy D, Wilson PW, Benjamin EJ, Larson MG, Kannel WB, Vasan RS (2002). Obesity and the risk of heart failure. N Engl J Med.

[37] Luo JW, Duan WH, Yu YQ, Song L, Shi DZ (2021). Prognostic Significance of triglyceride-glucose index for adverse cardiovascular events in patients with coronary artery disease: a systematic review and meta-analysis. Front Cardiovasc Med.

[38] Zhao Y, Sun H, Zhang W, Xi Y, Shi X, Yang Y, Lu J, Zhang M, Sun L, Hu D (2021). Elevated triglyceride-glucose index predicts risk of incident ischaemic stroke: the rural Chinese cohort study. Diabetes Metab.

[39] Barzegar N, Tohidi M, Hasheminia M, Azizi F, Hadaegh F (2020). The impact of triglyceride-glucose index on incident cardiovascular events during 16 years of follow-up: Tehran lipid and glucose study. Cardiovasc Diabetol.

[40] Guo W, Zhao L, Mo F, Peng C, Li L, Xu Y, Guo W, Sun A, Yan H, Wang L (2021). The prognostic value of the triglyceride glucose index in patients with chronic heart failure and type diabetes a retrospective cohort study. Diabetes Res Clin Pract.

[41] Yang S, Du Y, Liu Z, Zhang R, Lin X, Ouyang Y, Chen H (2021). Triglyceride-Glucose Index and Extracellular Volume Fraction in Patients With Heart Failure. Front Cardiovasc Med.

[42] Aroor AR, Mandavia CH, Sowers JR (2012). Insulin resistance and heart failure: molecular mechanisms. Heart Fail Clin.

[43] Li Z, Zhao H, Wang J (2021). Metabolism and chronic inflammation: the links between chronic heart failure and comorbidities. Front Cardiovasc Med.

[44] Nishida K, Otsu K (2017). Inflammation and metabolic cardiomyopathy. Cardiovasc Res.

[45] Fu Q (2019). Sex differences in sympathetic activity in obesity and its related hypertension. Ann N Y Acad Sci.

[46] Zhou MS, Schulman IH, Zeng Q (2012). Link between the renin-angiotensin system and insulin resistance: implications for cardiovascular disease. Vasc Med.

[47] Liu F, Ling Q, Xie S, Xu Y, Liu M, Hu Q (2023). Association between triglyceride glucose index and arterial stiffness and coronary artery calcification: a systematic review and exposure-effect meta-analysis. Cardiovasc Diabetol.

[48] Boyman L, Karbowski M, Lederer WJ (2020). Regulation of mitochondrial ATP production: Ca(2+) signaling and quality control. Trends Mol Med.

[49] Eisner DA, Caldwell JL, Trafford AW, Hutchings DC (2020). The control of diastolic calcium in the heart: basic mechanisms and functional implications. Circ Res.

[50] Thai PV, Tien HA, Van Minh H, Valensi P (2020). Triglyceride glucose index for the detection of asymptomatic coronary artery stenosis in patients with type 2 diabetes. Cardiovasc Diabetol.

[51] Lee SB, Ahn CW, Lee BK, Kang S, Nam JS, You JH, Kim MJ, Kim MK, Park JS (2018). Association between triglyceride glucose index and arterial stiffness in Korean adults. Cardiovasc Diabetol.

[52] Abdul-Ghani MA, Tripathy D, DeFronzo RA (2006). Contributions of beta-cell dysfunction and insulin resistance to the pathogenesis of impaired glucose tolerance and impaired fasting glucose. Diabetes Care.

[53] Wang X, Ni J, Guo R, Li L, Su J, He F, Fan G (2022). SGLT2 inhibitors break the vicious circle between heart failure and insulin resistance: targeting energy metabolism. Heart Fail Rev.

[54] Mohan M, Al-Talabany S, McKinnie A, Mordi IR, Singh JSS, Gandy SJ, Baig F, Hussain MS, Bhalraam U, Khan F (2019). A randomized controlled trial of metformin on left ventricular hypertrophy in patients with coronary artery disease without diabetes: the MET-REMODEL trial. Eur Heart J.

