# Supplementary material for: Prevalence estimates of the insulin resistance and associated prevalence of heart failure among United Status adults
Source: BMC Cardiovasc Disord. 2023 Jun 10;23:294. doi: 10.1186/s12872-023-03294-9 (PMC10257843; doi:10.1186/s12872-023-03294-9)
Supplement: Supplementary file 1 — Additional file 1: Table S1. Association of the triglyceride-glucose index with the prevalence of specific cardiovascular diseases in adult Americans from the Nation Health and Nutrition Examination Survey 2009-2018. Table S2. Sensitive analysis of the association between the triglyceride-glucose index and the prevalence of heart failure in adult Americans based on the multiple-imputation analysis. Table S3. Baseline characteristics after propensity score matching of adult Americans from the Nation Health and Nutrition Examination Survey 2009-2018. Table S4. The associations of the triglyceride-glucose index with the prevalence of heart failure in the propensity score matched from the Nation Health and Nutrition Examination Survey 2008-2018. Table S5. The associations of the triglyceride-glucose index with the prevalence of heart failure after adjusted hypoglycemic agents, and lipid-lowering drugs from the Nation Health and Nutrition Examination Survey 2008-2018. Figure S1. The weighted mean values of the triglyceride-glucose index in adult Americans from the Nation Health and Nutrition Examination Survey 2009-2018. A: The weighted mean values of the triglyceride-glucose index of different age stages; B: The weighted mean values of the triglyceride-glucose index of a different gender; C: The weighted mean values of the triglyceride-glucose index of different races. Bar graphs and error bars represent weighted mean ± SE. Figure S2. Risk factor association with the triglyceride-glucose index. Figure S3. The maybe underlying mechanism of insulin resistance-induced heart failure. [file 12872_2023_3294_MOESM1_ESM.docx]

**Prevalence Estimates of the Insulin Resistance and Triglyceride Glucose Index and Associated Risk of Heart Failure** **among Adults from the United States from 2009 to 2018: A cross-sectional study**

Xiaozhong Li1 M.D., Jihong Wang1 M.D., Liyan Niu1, M.D., Ziqi Tan1, M.D., Jianyong Ma2 M.D, Ling He3 M.D., Peng Yu4 M.D., Xiao Liu5,6, M.D. Juxiang Li1 M.D.

1 Department of Cardiovascular Medicine, the Second Affiliated Hospital of Nanchang University, 330006, Nanchang, China

2 Department of Pharmacology and Systems Physiology University of Cincinnati College of Medicine, Cincinnati, OH, 45267, Unite Status

3 Department of Geriatrics Medicine, the Second Affiliated Hospital of Nanchang University, 330006, Nanchang, China

4 Department of Endocrinology Medicine, the Second Affiliated Hospital of Nanchang University, 330006, Nanchang, China

5 Department of Cardiology, Sun Yat-sen Memorial Hospital of Sun Yat-sen University, Guangzhou, 510080, Guangdong, China.

6 Guangzhou Key Laboratory of Molecular Mechanism and Translation in Major Cardiovascular Disease, Guangzhou, 510120, Guangdong, China

**Table S1** **Association of the triglyceride-glucose index with the prevalence of specific cardiovascular diseases in adult Americans from the Nation Health and Nutrition Examination Survey 2009-2018**

**Table S2 Sensitive analysis of the association between the triglyceride-glucose index and the prevalence of heart failure in adult Americans based on the multiple-imputation analysis.**

**Table S3 Baseline characteristics after propensity score matching of adult Americans from the Nation Health and Nutrition Examination Survey 2009-2018**

**Table S4 The associations of the triglyceride-glucose index with the prevalence of heart failure in the propensity score matched from the Nation Health and Nutrition Examination Survey 2008-2018**

**Table S5 The associations of the triglyceride-glucose index with the prevalence of heart failure after adjusted hypoglycemic agents, and lipid-lowering drugs from the Nation Health and Nutrition Examination Survey 2008-2018**

**Figure S1 The weighted mean values of the triglyceride-glucose index in adult Americans from the Nation Health and Nutrition Examination Survey 2009-2018. A: The weighted mean values of the triglyceride-glucose index of different age stages; B: The weighted mean values of the triglyceride-glucose index of a different gender; C: The weighted mean values of the triglyceride-glucose index of different races. Bar graphs and error bars represent weighted mean ± SE.**

**Figure S2 Risk factor association with the triglyceride-glucose index.**

**Figure S3 The maybe underlying mechanism of insulin resistance-induced heart failure.**

**TABLE S1** **Association of the triglyceride-glucose index with the prevalence of specific cardiovascular diseases in adult Americans from the Nation Health and Nutrition Examination Survey 2009-2018**

| Disease | Odds ratio (95% confidence interval) | P-value |
| --- | --- | --- |
| CHD | 1.41 (1.07,1.83) | 0.020 |
| Hypertension | 1.56 (1.43,1.70) | <0.001 |
| Dyslipidemia | 2.48 (2.25,2.74) | <0.001 |
| Stroke | 1.07 (0.86,1.33) | 0.500 |

Note: The TyG index was included as a continuous variable in the binary-weighted logistic regression analysis, which was used to evaluate the association between TyG index levels and specific cardiovascular diseases, and the result was expressed as odds ratios and 95% confidence intervals with the pre-defined model. The model was adjusted for age, sex, race, BMI, current smoking, marital status, poverty level, and education status.

Abbreviations: CHD: coronary heart disease.

**Table S2 Sensitive analysis of the association between the triglyceride-glucose index and the prevalence of heart failure in adult Americans based on the multiple-imputation analysis.**

| Dataset item | B | SE | Odd ratio (95% confidence interval) | P |
| --- | --- | --- | --- | --- |
| 1 | 0.22 | 0.09 | 1.24 (1.04, 1.47) | < 0.001 |
| 2 | 0.22 | 0.09 | 1.24 (1.04, 1.47) | < 0.001 |
| 3 | 0.22 | 0.09 | 1.24 (1.04, 1.48) | < 0.001 |
| 4 | 0.22 | 0.09 | 1.25 (1.05, 1.49) | < 0.001 |
| 5 | 0.22 | 0.09 | 1.24 (1.04, 1.47) | < 0.001 |
| Pooled estimates | 0.22 | 0.09 | 1.24 (1.04,1.48) | 0.011 |

Note: multiple-imputation methods are based on 5 replications and the Markov-chain Monte Carlo method in the SAS MI procedure. Item 0 is raw data from NHANES, and items 1-5 are replenished data from the SAS MI procedure that used 5 replications and the Markov-chain Monte Carlo method based on BMI, LDL, eGFR, uric acid, hypertension, and dyslipidemia. The 5 replenished data were used to explore the association between the TyG index and HF. The TyG index was included as a continuous variable in the binary-weighted logistic regression analysis, which was used to evaluate the association between TyG index levels and HF, and the result was expressed as odds ratios and 95% confidence intervals with the pre-defined model. The model was adjusted for age, gender, race, marital, BMI, education status, LDL-C, eGFR, uric acid, diabetes mellitus, hypertension, dyslipidemia, stroke, CKD, INDFMMPC, moderate PA, sedentary, and current smoking. Then, pooled estimates from five imputed data.

Abbreviations: SE: Standard error; HF: heart failure; BMI: body mass index; TyG: triglyceride-glucose index; LDL-C: low-density lipoprotein cholesterol; CKD: chronic kidney dysfunction; INDFMMPC: family monthly poverty level category; eGFR: estimated glomerular filtration rate; PA: Physical activity. TyG index: triglyceride-glucose index; HF: heart failure. NHANES: Nation Health and Nutrition Examination Survey.

**Table S3 Baseline characteristics after propensity score matching of adult Americans from the Nation Health and Nutrition Examination Survey 2009-2018.**

| **Characteristics** | **Total** | **Quartiles of triglyceride-glucose index** | | P |
| --- | --- | --- | --- | --- |
|  |  | Q1-Q3 (<9.0) | Q4 (≥9.0) |  |
|  | 4933 | 2468 | 2465 |  |
| TyG index | 8.9 (0) | 8.4 (0) | 9.4 (0) | < 0.01 |
| Age, year | 52.0 (0.3) | 50.8 (0.5) | 52.4(0.4) | 0.17 |
| Female, n% | 2224 (45.1) | 1110 (45.0) | 1114 (45.2) | 0.93 |
| BMI, kg/m^2^ | 31.7 (0.1) | 31.7 (0.2) | 31.8 (0.2) | 0.63 |
| DBP, mm Hg | 72.0 (0.3) | 70.0 (0.4) | 72.0 (0.4) | < 0.01 |
| SBP, mm Hg | 126.0 (0.3) | 125 (0.4) | 127 (0.5) | < 0.01 |
| **Smoke status, n (%)** |  |  |  |  |
| Never smoke | 2476 (50.2) | 1251 (50.7) | 122 (49.7) |  |
| Former smoke | 2456 (49.8) | 1216 (49.3) | 1240 (50.3) |  |
| Current smoking | 1248 (25.3) | 629 (25.5) | 619 (25.1) |  |
| **Current alcohol drinking, n (%)** | 1207 (24.5) | 552 (22.4) | 655 (26.6) | 0.09 |
| **Race, n (%)** |  |  |  | 0.36 |
| Mexican American | 518 (10.5) | 244 (9.9) | 274 (11.1) |  |
| Other Hispanic | 293 (6.0) | 148 (6.0) | 145 (5.9) |  |
| Non-Hispanic White | 3354 (68.1) | 1666 (67.5) | 1691 (68.6) |  |
| Non-Hispanic Black | 449 (9.1) | 299 (12.1) | 150 (6.1) |  |
| Other Race | 313 (6.3) | 111 (4.5) | 202 (8.2) |  |
| **Marital status, n (%)** |  |  |  |  |
| Never married | 631(12.8) | 338 (13.7) | 293 (11.9) |  |
| Married | 2895 (58.7) | 1412 (57.2) | 1483 (60.2) |  |
| Other | 1406 (28.5) | 718 (29.1) | 688 (27.9) |  |
| **Education status, n (%)** |  |  |  | 0.42 |
| Primary school graduate or below | 1421(28.8) | 688 (27.9) | 730 (29.6) |  |
| Middle/high/special school | 2442 (49.5) | 1244 (50.4) | 1198 (48.6) |  |
| College graduate or above | 1075 (21.8) | 538 (21.8) | 537 (21.8) |  |
| **poverty level, n (%)** |  |  |  | 0.73 |
| Low | 1381(28.0) | 718 (29.1) | 664 (26.9) |  |
| Moderate | 666 (13.5) | 338 (13.7) | 328 (13.3) |  |
| High | 2885 (58.5) | 1411 (57.2) | 1474 (59.8) |  |
| **Physical activity, n (%)** |  |  |  | 0.76 |
| Moderate | 1904 (38.6) | 945 (38.3) | 961 (39.0) |  |
| Vigorous | 853 (17.3) | 469 (19.0) | 380 (15.4) |  |
| Sedentary/min | 401.1 (5.4) | 405.5 (8.4) | 396.6 (6.8) | 0.41 |
| **Laboratory results** |  |  |  |  |
| Total cholesterol, mmol/L | 5.2 (0.0) | 5.1 (0.0) | 5.3 (0.0) | < 0.01 |
| Triglycerides, mg/dL | 150.8 (1.9) | 96.5 (1.2) | 205.8 (1.9) | < 0.01 |
| HDL-C, mmol/L | 1.3 (0.0) | 1.4 (0.0) | 1.1 (0.0) | < 0.01 |
| LDL-C, mg/dL | 121.3 (0.5) | 121.2 (1.0) | 121.4 (1.0) | 0.85 |
| Fasting glucose, mg/dL | 114.8 (0.8) | 102.7 (0.4) | 127.0 (1.4) | < 0.01 |
| BUN, mmol/L | 5.2 (0) | 5.2 (0.1) | 5.2 (0.1) | 0.85 |
| Cr, mmol/L | 79.9 (0.6) | 80.2 (0.7) | 79.6 (0.8) | 0.63 |
| eGFR ml/min/1.73m^2^ | 125.0 (0.8) | 125.6 (1.8) | 124.4 (1.3) | 0.61 |
| uric acid, umol/L | 351.6 (1.4) | 349.7 (2.1) | 335.3 (2.3) | 0.28 |
| HbA1c | 5.9 (0) | 5.6 (0.0) | 6.2 (0.0) | < 0.01 |
| HOMA-IR | 1.5 (0) | 1.2 (0.0) | 1.9 (0.1) | < 0.01 |
| **Drugs, n (%)** |  |  |  |  |
| Lipid-lowering drugs | 2213 (44.9) | 931 (37.7) | 1282 (52.0) | < 0.01 |
| Hypoglycemic agents | 774 (15.7) | 187 (7.6) | 587 (23.8) | < 0.01 |
| **History of disease, n (%)** |  |  |  |  |
| Hypertension | 2577 (52.2) | 1281 (51.9) | 1296 (52.6) | 0.77 |
| Diabetes mellitus | 1332 (27.0) | 660 (26.7) | 672 (27.3) | 0.76 |
| CHD | 221 (4.5) | 91 (3.7) | 130 (5.3) | 0.06 |
| Stroke | 174 (3.5) | 86 (3.5) | 88 (3.6) | 0.84 |
| CKD | 158 (3.2) | 81 (3.2) | 117 (3.3) | 0.94 |
| Dyslipidemia | 2048 (61.8) | 1525 (61.8) | 1523 (61.8) | 0.99 |
| Heart failure | 184 (3.7) | 81 (3.3) | 103 (4.2) | 0.04 |

Note: Data are expressed as mean (SE) and numbers (percentage) as appropriate. All estimates were weighted to be nationally representative.

Abbreviations: SE: Standard error; TyG index: triglyceride-glucose index; BMI: body mass index; DBP: diastolic blood pressure; HDL-C: high-density lipoprotein cholesterol; LDL-C: low-density lipoprotein cholesterol; SBP: systolic blood pressure; HbA1c: glycated hemoglobin; BUN: urea nitrogen; Cr: creatinine, eGFR: estimated glomerular filtration rate; HOMA-IR: Homeostatic model assessment of insulin resistance; CHD: coronary heart disease, CKD: chronic kidney dysfunction.

**Table S4 The associations of the triglyceride-glucose index with the prevalence of heart failure in the propensity score matched from the Nation Health and Nutrition Examination Survey 2011-2016.**

| **TyG index** | **Cases/N** | **Crude Model**  **OR (95%CI)** | **P-value** |
| --- | --- | --- | --- |
| Per 1 unit increase | 230/4733 | 1.52 (1.17,1.98) | < 0.01 |
| Categories |  |  |  |
| Q1-Q3 (<9.0)) | 111/2468 | Ref. | 1.0 |
| Q4 (≥ 9.0) | 119/2465 | 1.31 (0.98, 1.78) | 0.08 |

**Table S5** **The associations of the triglyceride-glucose index with the prevalence of heart failure after adjusted hypoglycemic agents, and lipid-lowering drugs from the Nation Health and Nutrition Examination Survey 2008-2018**

| **TyG index** | | **Cases/N** | | **Model 1**  **OR (95%CI)** | | **P-value** | | **Model 2**  **OR (95%CI)** | | **P-value** |
| --- | --- | --- | --- | --- | --- | --- | --- | --- | --- | --- |
| Per 1 unit increase | | 342/12388 | | 1.34 (1.02,1.76) | | 0.04 | | 1.72 (1.15,2.58) | | 0.01 |
| Quartiles |  | |  | |  | |  | |  |  |
| Q1 (< 8.12) | | 61/3040 | | Ref. | | 1.0 | | Ref. | | 1.0 |
| Q2 (8.12- 8.55) | | 94 /3133 | | 0.91 (0.52,1.56) | | 0.73 | | 0.66 (0.19,2.26) | | 0.51 |
| Q3 (8.55- 9.0) | | 93/3113 | | 1.13 (0.71,1.80) | | 0.59 | | 1.04 (0.34,3.18) | | 0.94 |
| Q4 (≥ 9.0) | | 155/3102 | | 1.45 (0.87,2.41) | | 0.16 | | 1.45 (0.57,3.36) | | 0.44 |
| P for trend | |  | | < 0.001 | |  | | < 0.001 | |  |
| Categories |  | |  | |  | |  | |  |  |
| Q1-Q3 (< 9.0)) | | 279/9286 | | Ref. | | 1.0 | | Ref. | | 1.0 |
| Q4 (≥ 9.0) | | 155/3102 | | 1.40 (1.02,1.90) | | 0.04 | | 1.61 (0.88,2.95) | | 0.05 |

Note: Model 1was adjusted for age, gender, race, marital status, BMI, education status, LDL-C, eGFR, uric acid, diabetes mellitus, hypertension, dyslipidemia, stroke, CKD, INDFMMPC, moderate PA, sedentary, current smoking. Model 2 was adjusted for Model 1, hypoglycemic agents, and lipid-lowering drugs.

Abbreviations: 95% CI: 95% confidence interval; HF: heart failure; TyG index: triglyceride-glucose index; OR: odds ratio; BMI: body mass index; LDL-C: low-density lipoprotein cholesterol, eGFR: estimated glomerular filtration rate; CKD: chronic kidney dysfunction; INDFMMPC: family monthly poverty level category, PA: Physical activity.


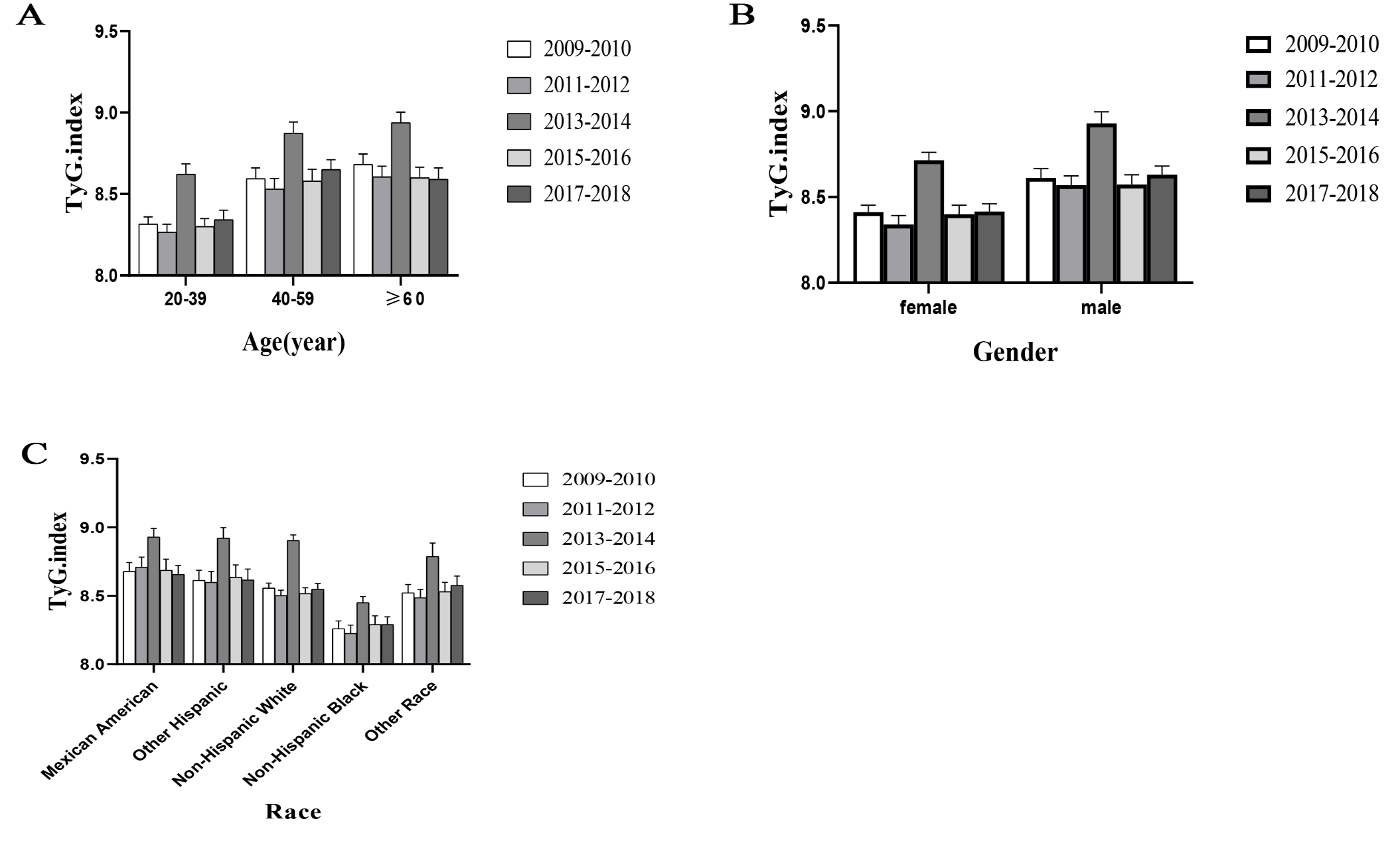


**Figure S1** **The weighted mean values of the triglyceride-glucose index in adult Americans from the Nation Health and Nutrition Examination Survey 2009-2018**. A: The weighted mean values of the triglyceride-glucose index of different age stages; B: The weighted mean values of the triglyceride-glucose index of a different gender; C: The weighted mean values of the TyG index of different races. Bar graphs and error bars represent weighted mean ± SE.

Abbreviations: TyG: triglyceride-glucose index; SE: standard error.


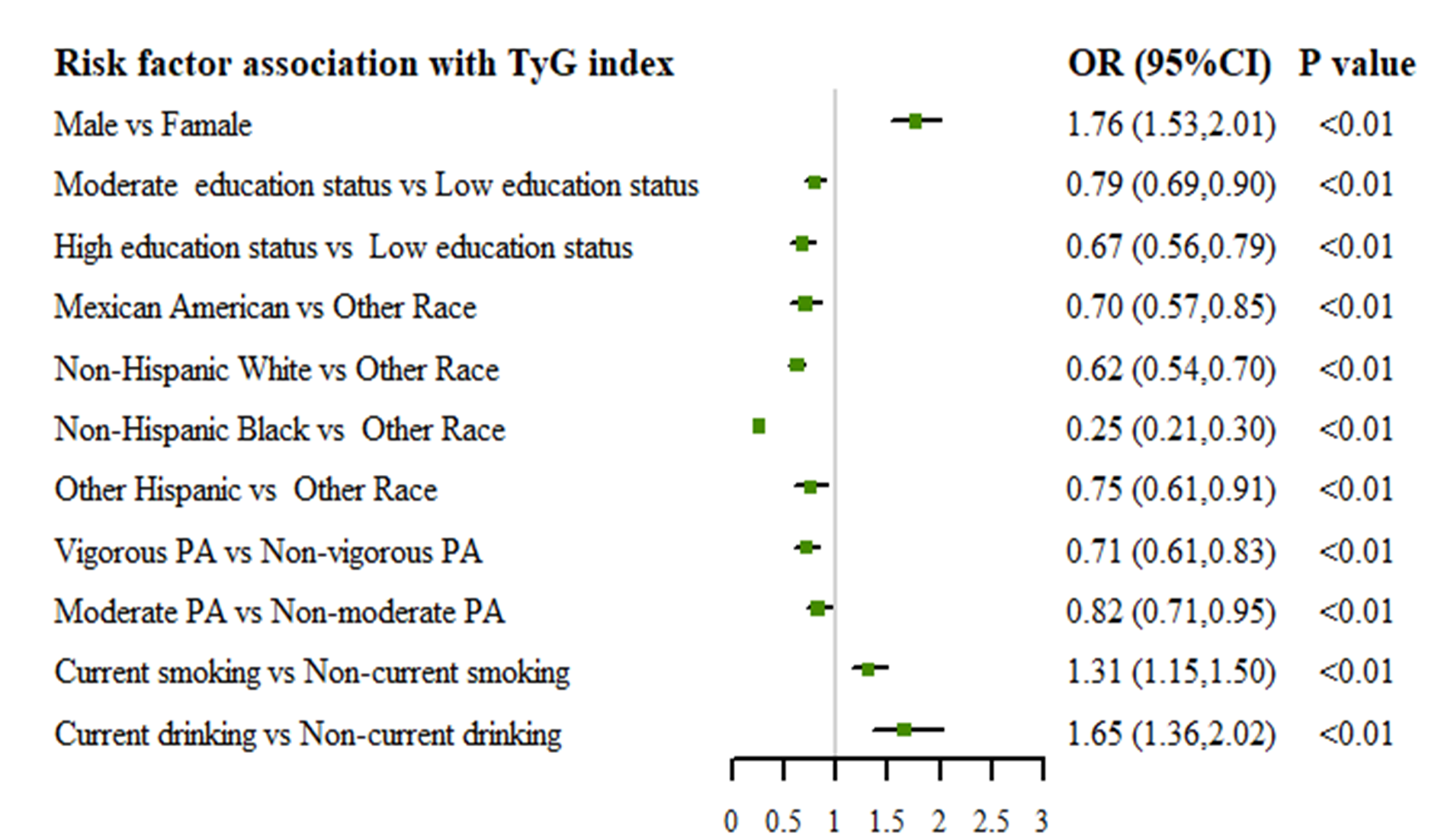


**Figure S2 Risk factor association with the triglyceride-glucose index.**

Abbreviations: low education status: Primary school graduate or below; moderate education status: Middle/high/special school; higher education status: College graduate or above; PA: Physical activity.

**
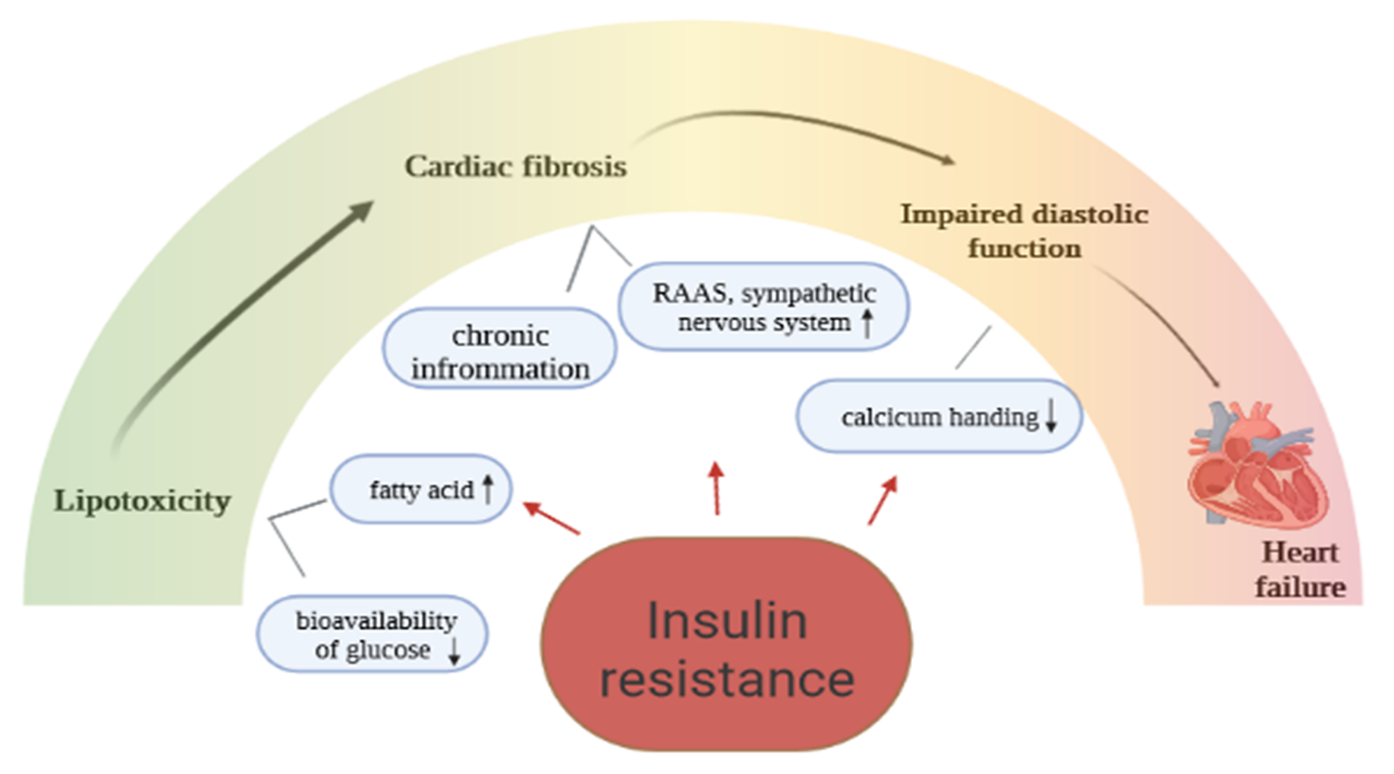
**

**Figure S3** The maybe underlying mechanism of insulin resistance-induced heart failure.

## Abbreviations: Renin-Angiotensin-Aldosterone System
